# The major effects of health-related quality of life on 5-year survival prediction among lung cancer survivors: applications of machine learning

**DOI:** 10.1038/s41598-020-67604-3

**Published:** 2020-07-01

**Authors:** Jin-ah Sim, Young Ae Kim, Ju Han Kim, Jong Mog Lee, Moon Soo Kim, Young Mog Shim, Jae Ill Zo, Young Ho Yun

**Affiliations:** 1 Department of Biomedical Science, Seoul National University College of Medicine, Seoul, Korea; 2 National Cancer Control Institute, National Cancer Center, Goyang, Korea; 3 Department of Biomedical Informatics, Seoul National University College of Medicine, Seoul, Korea; 4 Center for Lung Cancer, National Cancer Center, Goyang, Korea; 5 Lung and Esophageal Cancer Center, Samsung Comprehensive Cancer Center, Samsung Medical Center, Seoul, Korea; 6 Department of Family Medicine, Seoul National University College of Medicine, Seoul, Korea

**Keywords:** Health care, Medical research, Oncology, Risk factors, Signs and symptoms

## Abstract

The primary goal of this study was to evaluate the major roles of health-related quality of life (HRQOL) in a 5-year lung cancer survival prediction model using machine learning techniques (MLTs). The predictive performances of the models were compared with data from 809 survivors who underwent lung cancer surgery. Each of the modeling technique was applied to two feature sets: feature set 1 included clinical and sociodemographic variables, and feature set 2 added HRQOL factors to the variables from feature set 1. One of each developed prediction model was trained with the decision tree (DT), logistic regression (LR), bagging, random forest (RF), and adaptive boosting (AdaBoost) methods, and then, the best algorithm for modeling was determined. The models’ performances were compared using fivefold cross-validation. For feature set 1, there were no significant differences in model accuracies (ranging from 0.647 to 0.713). Among the models in feature set 2, the AdaBoost and RF models outperformed the other prognostic models [area under the curve (AUC) = 0.850, 0.898, 0.981, 0.966, and 0.949 for the DT, LR, bagging, RF and AdaBoost models, respectively] in the test set. Overall, 5-year disease-free lung cancer survival prediction models with MLTs that included HRQOL as well as clinical variables improved predictive performance.

## Introduction

Globally, lung cancer has been the most common cancer for several decades^1^. Due to advances in early detection and improved treatment strategies^1,2^, lung cancer mortality has decreased worldwide^3^, and Korean age-adjusted lung cancer mortality has decreased by 3.4% annually since 2012^4^. With this increase in the number of cancer survivors, it has become important to classify these individuals into precise prognostic groups and provide them with appropriate information for better follow-up planning and personalized self-management.^5^

Many lung cancer survivors have reported that they had diverse health difficulties^2,6^, and their health function or symptom burden was more severe than that of others^6^. In fact, many recent studies have suggested that patient-reported outcomes (PROs), such as health-related quality of life (HRQOL) or clinical data, can provide clear prognostic information^7,8^. In our previous study of disease-free lung cancer survivors^9,10^, we found that several HRQOL variables showed prognostic potential, and thus, HRQOL or lifestyle factors can be used to identify patients who could benefit from a specific intervention. Therefore, we aimed to predict lung cancer survivors’ disease-free 5-year survival after primary treatment for lung cancer ended, i.e., the patient survived without any signs or symptoms of that cancer, such as local or regional relapses of the tumor or development of distant metastases, using a combination of sociodemographic, clinical and HRQOL variables.

In general, statistical approaches focus on inferring the characteristics of a population from sample data^11^, while machine learning techniques (MLTs) may focus on predicting future values by analyzing the given data and have the potential to maximize the prediction accuracy of large clinical data sets^12^. In addition, MLTs are more suitable for developing prediction models with dozens of parameters when more prognostic variables are included, because standard statistics do not generally work in this situation^13^. However, although a variety of prediction models based on MLTs for cancer mortality have been developed and utilized in clinical settings^14,15^, there have been fewer studies regarding the development of MLT-based lung cancer survival prediction models using HRQOL factors.

Here, we proved that the machine learning model including HRQOL data in addition to demographic and clinical parameters was more predictive than existing models that include only demographic and clinical characteristics. We compared the performance of five MLTs by applying each of them to feature set 1 (in which the model considers only demographic and clinical characteristics) and feature set 2 (in which HRQOL factors are added to the variables from feature set 1). The five MLTs used are as follows: decision tree (DT), logistic regression (LR), bagging, random forest (RF), and adaptive boosting (AdaBoost).

## Results

### Data proportions after data up-sampling and splitting

If the data were well sampled and solved the imbalance problem well, there should be no statistically significant differences in the final comparison of sociodemographic and clinical variables between the deceased and living groups based on the up-sampled data. The final comparison of sociodemographic and clinical variables between the deceased and living groups based on the up-sampled data is shown in Table 1. No statistically significant differences between the deceased and living groups were found after balancing. After missing data imputation and data balancing, the data were split into a training set (80%, n = 1,140) and a validation set (20%, n = 286). There were no significant differences between the training and validation sets.

**Table 1:** Comparison of the baseline characteristics between the living and deceased groups with up-sampled data.

Variable	Balanced up-sampled data				
	Living (N = 713)		Deceased (N = 713)		p-value
	n %		n %		
**Age (years)**	62.51 ± 8.55		66.21 ± 8.31		< 0.001
< 65	393	63.3	228	36.7	< 0.001
≥ 65	320	39.8	485	60.2	
**Sex**					
Female	177	69.4	78	30.6	< 0.001
Male	537	45.8	635	54.2	
**Monthly income (USD)**					
≥ 3,000	207	69.5	91	30.5	< 0.001
< 3,000	506	44.9	622	55.1	
**Education**					
≥ High school degree	185	56.4	143	43.6	0.01
< High school degree	528	48.1	570	51.9	
**Currently married**					
Yes	655	50	656	50	0.92
No	58	50.4	57	49.6	
**FEV1/FVC**	72.55 ± 15.11		65.77 ± 10.62		< 0.001
(FEV1/FVC)*100 ≥ 0.7	454	61.7	282	38.3	< 0.001
(FEV1/FVC)*100 < 0.7	259	37.5	431	62.5	
**Local tumor invasion**					
No	253	62.3	153	37.7	< 0.001
Yes	460	45.1	560	54.9	
**Regional lymph node metastasis**					
No	508	53.2	446	46.8	< 0.001
Yes	205	43.4	267	56.6	
**Stage**					
Stage 0–I	464	56.9	352	43.1	< 0.001
Stage II–III	249	40.8	361	59.2	
**Recurrence**					
No	630	62.8	373	37.2	< 0.001
Yes	83	19.6	340	80.4	
**Number of comorbidities**					
0	320	49	333	51	0.49
≥ 1	393	50.8	380	49.2	
**Treatment type**					
OP	435	51.7	417	48.6	< 0.001
OP + RT	41	37.6	68	62.4	
OP + CT	193	53.6	167	46.4	
OP + CT + RT	44	40	66	60	
**Time since diagnosis**	2.93 ± 1.59		2.983 ± 1.68		0.29
≥ 3 years	307	53	272	47	0.06
< 3 years	406	47.9	441	52.1	

OP, operation; RT, radiation therapy; CT, chemotherapy; FEV1/FVC, forced expiratory volume 1/forced vital capacity.

### Importance of the prognostic factors included in the developed prediction model

The importance of the selected prognostic variables was compared with MLT Table 2. The calculated mutual variable importance was normalized, and the sum ranged between 0 and 100%. In feature set 1, cancer stage (II–III) was identified as the most important factor in the DT, bagging, and AdaBoost models. Age was identified as the most important factor in the LR and RF models. In feature set 2, appreciation of life was identified as the most important factor in the DT model, while cancer stage and body mass index (BMI) (kg/m^2^) before the operation were most important in the bagging model, and sex and anxiety were most important in the RF model. Regional lymph node metastasis and dyspnea were the most important predictors in the AdaBoost model, and personal strength was the most important predictor in the LR model.

**Table 2:** The normalized importance scores of prognostic variables for each of the five MLTs.

Domain	Variable	Feature sets									
		Feature set 1: sociodemographic and clinical variables					Feature set 2: PRO variables added to feature set 1				
		Normalized variable importance (%)					Normalized variable importance (%)				
		Model	Model	Model	Model	Model LR*	Model	Model	Model	Model	Model LR*
		DT	Bagging	RF	AdaBoost		DT	Bagging	RF	AdaBoost	
Clinical factors	Cancer stage II–III	**24.36**	**20.06**	19.39	**23.57**	23.44	**9.78**	**7.49**	6.06	6.60	**11.40**
	Local invasion of tumor	8.90	14.34	14.28	10.66	12.50	**8.20**	6.31	5.58	3.26	NS
	Regional lymph node metastasis	23.71	10.25	10.42	9.13	NS	**8.59**	6.20	6.42	**7.58**	NS
Sociodemographic factors	Low household income (< 3,000$)	13.82	18.46	16.00	14.26	20.49	4.93	5.33	5.29	5.60	5.14
	Age over 65 years	20.45	19.87	**21.87**	23.19	**26.75**	6.04	6.28	**7.40**	**7.02**	**11.61**
	Male	8.76	17.01	18.03	19.19	24.84	5.90	5.70	**7.61**	6.96	**11.36**
HRQOL factors	BMI (kg/m^2^) before the operation (≥ 23. 5)						5.91	**7.38**	**6.95**	6.55	10.09
	Anxiety						3.34	5.49	**7.12**	6.28	7.04
	Depression						3.59	**6.37**	6.60	6.36	4.46
	Poor physical functioning						1.74	2.36	1.74	1.48	6.47
	Role functioning						1.64	1.73	1.53	2.14	3.54
	Poor dyspnea						5.47	5.89	6.59	**7.51**	3.82
	Poor appetite loss						3.53	4.24	3.35	4.44	NS
	Poor diarrhea						1.95	2.64	2.10	2.78	NS
	Poor lung cancer-specific cough						3.00	4.27	3.98	4.28	NS
	Poor pain in chest						3.36	4.41	4.34	3.88	NS
	Low new possibility						5.93	5.26	4.88	3.69	7.69
	Low personal strength						6.62	5.45	5.56	6.11	**12.23**
	Low appreciation of life						**10.48**	**7.21**	6.89	**7.47**	5.19

NS, nonsignificant; BMI, body mass index; HRQOL, health-related quality of life; DT, decision tree; RF, random forest; LR, logistic regression.

*LR variable selection using stepwise feature selection with a 5% significance level.

The most important variable in the top 20% from each model are highlighted in bold font.

### Comparisons of the MLT-based models’ performances

Based on the accuracy of the prediction model with cross-validation, each MLT-based prediction model’s performance was measured. The parameters used in each lung cancer survival prediction model are summarized in Table 3, including the validation method with N folds, the training and testing set sizes, the tuning parameter, the performances of the classifiers on the testing set, and the validation results from the fivefold cross-validation dataset using two different feature sets. Among the overall model performances for feature set 1, there were no significant differences in the model accuracies (ranging from 0.647 to 0.713),the LR model had the lowest accuracy, and the RF model had the highest accuracy in feature set 2 (0.746 and 0.916, respectively). Among the models for the fivefold cross-validation sets, the test accuracy of the AdaBoost model exceeded those of the other prognostic models (0.745, 0.825, 0.773, 0.941, and 0.948 for the DT, LR, bagging, RF and AdaBoost models, respectively) in feature set 2.

**Table 3:** Model comparisons based on the five machine leaning techniques.

Feature set	Machine learning algorithm	Validation method	N folds	Training set size	Testing set size	Training accuracy	Testing accuracy
1	DT	Holdout sampling		1,140	286	0.668	0.703
	DT	Cross-validation	5	912	286	0.625	0.692
	LR	Holdout sampling		1,140	286	0.663	0.647
	LR	Cross-validation	5	912	286	0.657	0.632
	Bagging	Holdout sampling		1,140	286	0.680	0.710
	Bagging	Cross-validation	5	912	286	0.655	0.706
	RF	Holdout sampling		1,140	286	0.675	0.713
	RF	Cross-validation	5	912	286	0.675	0.692
	AdaBoost	Holdout sampling		1,140	286	0.668	0.696
	Real AdaBoost	Cross-validation	5	912	286	0.642	0.713
2	DT	Holdout sampling		1,140	286	0.780	0.762
	DT	Cross-validation	5	912	286	0.758	0.745
	LR	Holdout sampling		1,140	286	0.791	0.746
	LR	Cross-validation	5	912	286	0.814	0.825
	Bagging	Holdout sampling		1,140	286	0.976	0.930
	Bagging	Cross-validation	5	912	286	0.794	0.776
	RF	Holdout sampling		1,140	286	0.949	0.916
	RF	Cross-validation	5	912	286	0.918	0.941
	AdaBoost	Holdout sampling		1,140	286	0.943	0.878
	Real AdaBoost	Cross-validation	5	912	286	0.932	0.948

DT, decision tree; RF, random forest; LR, logistic regression.

Feature set 1 includes sociodemographic and clinical variables.

Feature set 2 includes PRO variables and the variables included in feature set 1.

**Figure 1:**
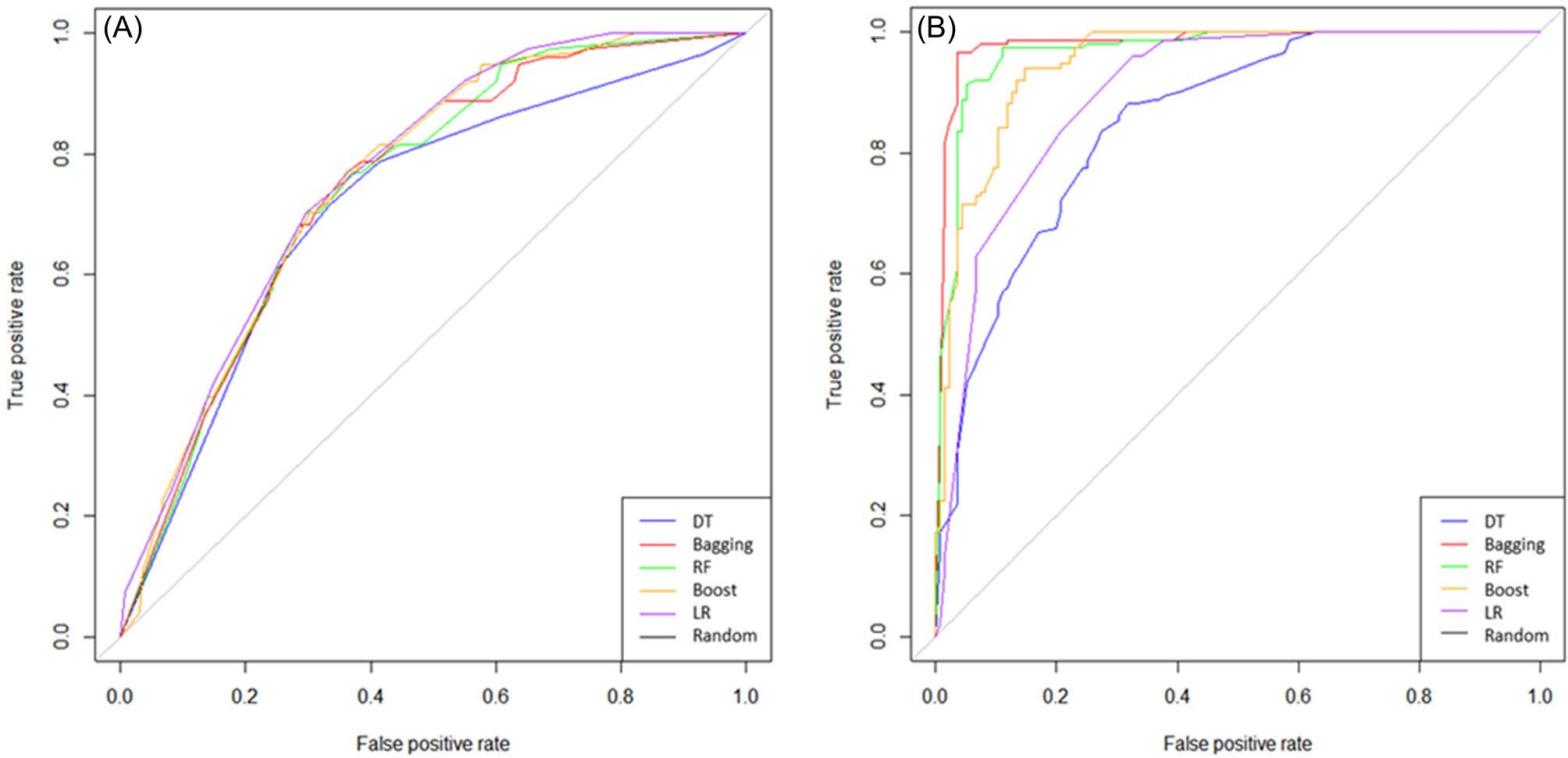
Comparison of ROC curves for the five MLT-based lung cancer models using the cross-validation test set. DT, decision tree; RF, random forest; Boost, AdaBoost; LR, logistic regression. (**A**) Model from feature set 1, (**B**) Model from feature set 2.

**Figure 2:**
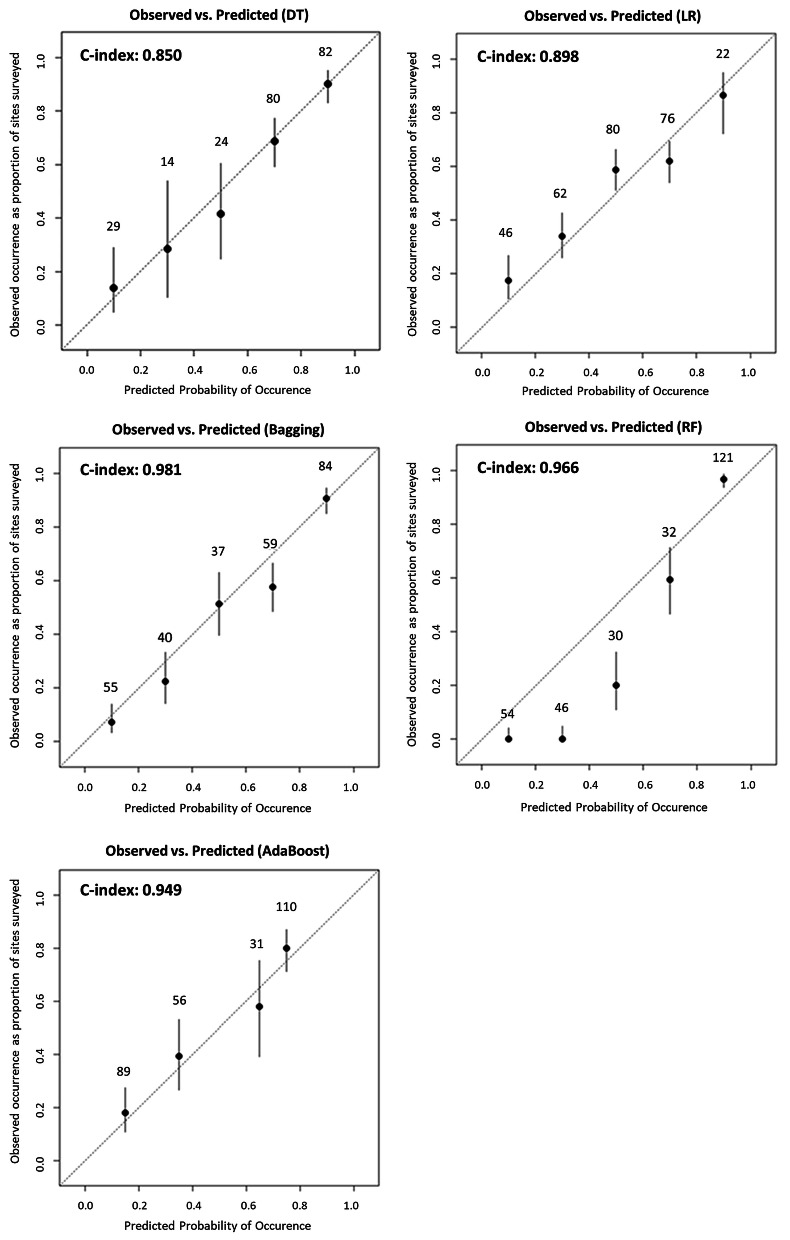
Calibration plots for each MLT-based lung cancer model at five risk levels using the cross-validation test set. DT, decision tree; RF, random forest; LR, logistic regression.

The receiver operating characteristic (ROC) curves for each feature set of the 5 MLT models based on the cross-validation set that were used to calculate the area under the curve (AUC) were also drawn Fig. 1. Among the models based on feature set 2, the bagging model outperformed the other prognostic models (AUC = 0.850, 0.898, 0.981, 0.966, and 0.949 for the DT, LR, bagging, RF and AdaBoost models, respectively) in fivefold cross-validation. Figure 2 shows the prediction values on the x-axis, and the H-statistic is plotted as five groups in the calibration graphs from the testing set. Four of the models’ calibration plots aligned well with the diagonal lines, although that of the AdaBoost model did not.

## Methods

### Data acquisition

We first identified 2,049 participants aged over 18 years who underwent primary lung cancer surgery between 2001 and 2006 from the Samsung Medical Center or the National Cancer Center in South Korea cancer registries^16^. The participants were eligible if they (1) were diagnosed with lung cancer (stage 0–III), (2) were treated with curative surgery, and (3) had no evidence of a history of other cancer. We contacted eligible subjects by telephone, and those who agreed to participate were surveyed with the help of our staff at home or in the clinic. In this analysis, we also excluded subjects whose cancer had recurred at that time. As video-assisted thoracic surgery was not often performed from 2001 to 2006, we also excluded patients who received it. Thus, all patients in this study underwent pulmonary resection through open thoracotomy. A total of 1,633 survivors were pathologically diagnosed as disease-free and did not receive any treatment while the study was in progress,906 survivors completed the self-reported survey. After excluding patients with recurring cancer and those for whom the questionnaire was missing, 836 lung cancer survivors were initially included.

Lung cancer patients who did not have evidence of recurrence or death were censored at the last follow-up before the target date. In this study, a regular follow-up was undertaken for each patient based on each hospital’s registry after the completion of treatment. If a patient died during the follow-up, the family caregivers were asked the date of death. Among 836 patients, we excluded 27 subjects whose survival status was censored by December 31, 2011. In total, 809 patients were included in this study. Ethics approval was obtained from the Institutional Review Boards of the National Cancer Center and Samsung Medical Center. The patients eligible to participate were asked to provide informed consent to the staff. Written informed consent was obtained from all the participants before the study. The current study inclusion followed the ethical standards declared in the 1964 Declaration of Helsinki and its later amended version.

### Phased feature sets with selected prognostic factors

The study participants’ data included clinical information regarding the primary cancer site, date of cancer diagnosis, cancer stage, treatment type, and other clinical characteristics for all lung cancer survivors. Measuring patients’ symptoms or PROs with a self-reported questionnaire has high validity because asking people directly allows us to reliably obtain their symptom status, and the results can be replicated. Therefore, we collected HRQOL data as well among disease-free lung cancer survivors who were treated with primary lung cancer surgery and survived without cancer recurrence for more than one year through a self-reported questionnaire. Each participant completed the survey including important lung cancer survivorship issues such as HRQOL, anxiety, depression, and posttraumatic growth.

To increase the robustness and validity of a model during the process of prediction modeling, the selection of candidate predictors is important. Final candidate variables that met both the literature review evidence level and statistical significance based on univariate analyses from a previous study^16^ were selected. (Supplementary Table 1). The variables included demographics (age and sex), socioeconomic status (marital status, educational level, and monthly family income), and past medical history (cancer stage, local invasion of tumor, regional lymph node metastasis, recurrence, comorbidities, treatment type, and time since diagnosis). In addition, lifestyle factors such as BMI and metabolic equivalents of task (MET)-hours per week for physical activity (PA) were considered. For HRQOL assessment, the European Organization for Research and Treatment of Cancer Quality of Life Questionnaire Core 30 (EORTC QLQ-C30)^17^^,^ the Hospital Anxiety and Depression Scale (HADS)^18^, and the Posttraumatic Growth Inventory (PTGI) were also selected.

In our model development process, we also calculated a variance inflation factor (VIF) to detect multicollinearity (with a criterion of VIF score greater than 10) in our machine learning models. However, in our data, there were no variables with scores greater than 5, which indicates high correlation in the model; thus, we did not exclude any additional variables from our final candidate variables. In addition, because recurrence was also regarded as an outcome variable, we did not include that variable in the modeling process. For final model construction, we grouped the candidate prognostic factors into two feature sets: (1) sociodemographic and clinical factors and (2) a combination of PROs and lifestyle factors added to the variables from feature set 1.

**Figure 3:**
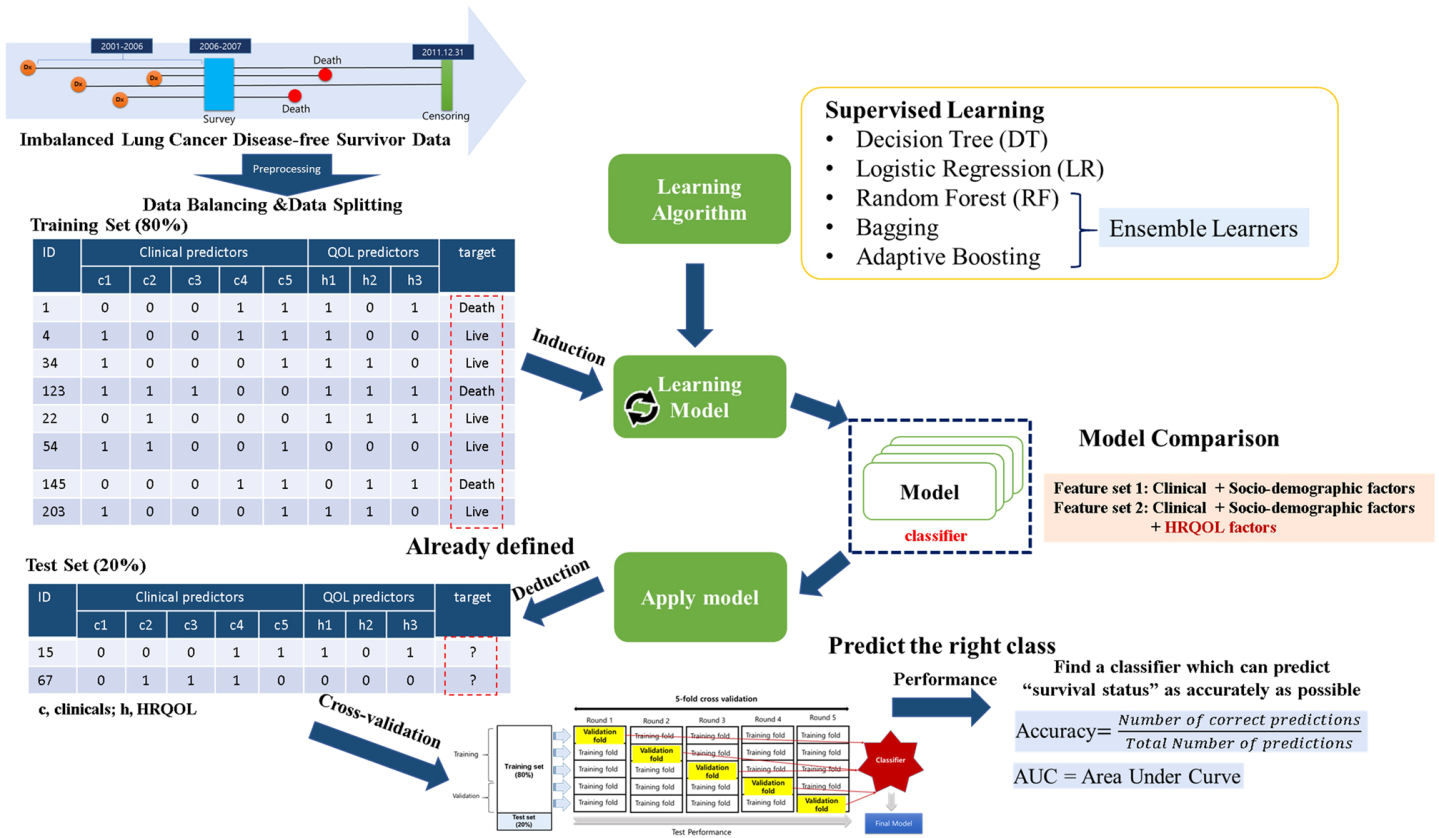
Study hypothesis and process.

We defined the endpoint as the fifth year from the date of survey completion after primary treatment for lung cancer ended or the date of any cause of death. To obtain the date of overall survival (OS) after survey completion, we used the National Statistical Office death database linkage through December 31, 2011, as an outcome measure of any cause of death. During the follow-up, we identified 96 deaths (11.9%) and 713 (89.1%) survivals among the 809 subjects. The study design and process is shown in Fig. 3, and the study flow is shown in Supplementary Figure 1.

### Statistical analyses

#### Data preprocessing

Most machine learning algorithms only induce knowledge from the given data, the quality of the extracted knowledge can be determined by the quality of the background data^19^. Therefore, we first attempted to impute missing values based on important information available in the data set. Although several methodologies are used to treat missing values, we applied the k-nearest neighbor (KNN) algorithm to estimate and substitute our missing data. The KNN algorithm is useful because it can predict both binary and continuous features together. Prior to imputing the missing values, we first investigated the missing numbers of each variable. Then, using the R package “DMwR” and the function “Knn Imputation”, we replaced and imputed the weighted average numbers of the nearest 5 neighbors (k = 5) with missing HRQOL values in our algorithm. The “before” and “after” missing values plotting are shown in the Supplementary Figure 2. The red points are the proportion of missing values, and we could observe that after KNN imputation, there were no more missing values in this data.

Then, to maximize the differences in prognostic strength of the HRQOL scores, we dichotomized each scale of the EORTC QLQ-C30 and EORTC QLQ-LC13 based on the score for the problematic group to investigate clinically meaningful differences: ≤ 33 on a scale of 0–100 for global QOL or functioning scale, and > 66 for symptom scale.^16,20^ In addition, we used the HADS dichotomized with a cut-off point of 8 (a borderline case of anxiety or depression) as the outcome measure.^21^ For PTGI, we dichotomized each variable according to the standardized manual^22^.

In the machine learning classification, important differences in proportions cannot show accurate predictions but also can lead to misleading results. Therefore, in order to make them allowable in real clinical settings, we had to deal with unbalanced problems when the value of finding a few ‘deceased’ classes were much higher than finding a majority. For this preprocessing, we used oversampling to reduce the error costs for the imbalanced data^23^^,^ and all our study results were based on oversampling. The imbalance in the distribution fails most algorithms from finding a proper solution. The number of ‘deceased’ and ‘living’ cases after oversampling was the equivalent of 713 ‘deceased’ (50%) and ‘living’ (50%) cases. Then, the holdout method was used to randomly split the data sample into two mutually exclusive training (80%) and testing (20%) sets. The training set was utilized to generate the prediction models, and the remaining data were employed as a testing set to estimate the models’ predictive performances.

#### Machine learning algorithms

Five supervised MLT-based classification models were trained to build each multivariable model to predict the 5-year survival rates for Korean lung cancer long-term survivors in the training set. DT, LR, RF and ensemble learning techniques such as bagging or AdaBoost were selected for the predictive feature selection process. The performances of the derived predictive models based on MLTs were internally validated by fivefold cross-validation.

##### Individual model learners

The DT and LR models were used for individual model learning. The main components of a DT model are nodes and branches, and the most important steps in building a model are splitting, stopping, and pruning^24^. In splitting, the purity of the resultant child nodes is used to choose between different potential input variables^25,26^. A well-classified prediction model shows a higher information gain, and the splitting procedure continues until the stopping criteria are met. Then, we pruned the training set at a point that improved the accuracy of the overall classification and increased the validation error. Each pruning step of the “cp” model can be calculated and plotted as a figure. Finally, DT models from the candidate feature set were developed.

LR is based on a logistic function that estimates the regression equation for a binary (0/1) dependent variable (classification problem). The value of the logit function can be inversely multiplied by the probability of success for the dependent variable so that the survival forecast can be applied to the classification problem. There is a growing perception that simultaneous evaluation of multiple exposures can reduce false-positive findings through several selection methods^27^. Therefore, we used stepwise selection for the LR model to select the most informative variables. The variable selection was performed in both directions, adding independent variables (forward) and removing previously added variables that were no longer influential (backward). In this stepwise selection process, a sequence of models starting with the null model and ending with the full model was derived. A 5% significance level was chosen as a threshold for the inclusion of a model variable. In this process, we used the generalized linear model (GLM) library from R-3.5.2.

##### Ensemble feature learners

Ensemble learners such as RF, bagging and adaptive boosting were utilized. Ensemble methods classify data by combining the results of multiple learners to improve classification accuracy by combining predictions from multiple classifiers. Bagging is a technique for generating a large number of training sets by resampling the given learning data with replacement^28^. With bagging, after generating multiple bootstrap samples, several predictive models are trained for each sample set, and then, the results of each model are combined and predicted. The RF model is a model that adds a random subspace to the bagging^29^. The difference between a RF model and bagging is that randomly, after choosing ‘m’ variables from among all the variables, the optimal classifier is found by using the ‘m’ selected variables. Finally, the AdaBoost model combines several weak learning algorithms to create a good classification model^30^. The AdaBoost model sequentially trains the classifiers to complement the weak points of the previous classifier. The R packages caret, randomForest, ipred, and adabag were used for ensemble learning.

#### Cross-validation

Compared to other models, models built with ensemble techniques are less likely to be overfitted, but it is still something to avoid. Tuning model parameters is one method to prevent overfitting, but it is not the only one. Training features are more likely to lead to overfitting than the model parameters, especially in ensemble learning. Therefore, having a reliable method to check the developed model for overfitting is more important. The choice for the best model based on the k-fold cross-validation results will lead to a model that is not overfitted, which is not necessarily the case for other issues, such as the out-of-bag error. The classifiers of each of the 10 models were trained and evaluated by fivefold cross-validation through the caret package in R.

#### Model discrimination and calibration

The training and test performance of the MLT algorithms were compared with the model accuracy, which is the proportion of correctly classified samples among the total data. Each predictive performance from the fivefold cross-validation was assessed according to the AUC. The AUCs and 95% confidence intervals (CIs) were calculated to compare the performances of all the proposed models (10 MLT models: 5 from set 1 and 5 from set 2). The normalized variable importance method (VIM)^31^ was used to determine the importance of explanatory prognostic variables in each of the 10 prediction models. Finally, to estimate the clinical discriminatory capacity of our fivefold cross-validated data sets, we divided the patients into 5 subgroups according to the calculated predictive scores and compared the predictive survival rates to the real-world of survival rates.

## Discussion

In this study, we demonstrated the major effects of HRQOL measurements in predicting survival among patients with disease-free lung cancer employing MLT ensemble learners. We also suggested that MLT-based survival prediction models could be used to assist conventional tools in predicting disease-free survivors’ clinical outcomes for lung cancer^32^ and monitoring their medical status instead of traditional prediction models with individual classification learner-based prediction features^16,33^. Therefore, we first developed 10 models (5 constructed from only clinical and sociodemographic variables and 5 constructed from clinical or sociodemographic variables and HRQOL data) based on five MLT models (individual learners: DT and LR,ensemble learners: RF, bagging, and AdaBoost) and then compared and validated their prognostic accuracies. Finally, each of the five models based on feature set 2 showed moderately good discrimination and well-calibrated performance compared to the models constructed from only clinical and sociodemographic variables.

Ensemble learning methods showed significantly greater model accuracies (more than 90%) than others in terms of AUC. These machine learning-based lung cancer survival prediction models are the first models developed with not only clinical or sociodemographic factors but also integration of information from multiple factors, such as HRQOL factors combined with clinical factors, ensuring better model performance in terms of both discrimination and calibration and even greater predictive ability than other machine learning models. Among the various MLTs, the RF and AdaBoost models proved superior to the other algorithms.

There are some possible explanations for the findings of this study. First, lung cancer survivors’ HRQOL plays a key role in survival in conjunction with assessments of clinical outcomes, including those based on MLTs. From systematic reviews, we found that there were impressive numbers of studies that showed a positive association between HRQOL and cancer survival. Based on this theoretical background, we constructed a new feature set, which included both clinical variables and HRQOL factors, that quantified good predictive accuracy in our data with five MLTs (DT, LR, bagging, AdaBoost, and RF). From the diverse MLT features, dyspnea, appreciation of life, BMI, anxiety and depression were selected as important variables in addition to cancer stage and sex. Additionally, it is possible that physical function^34–37^, dyspnea^16,38–42^, fatigue^34,36,40,43^, cough^40^^,^ anxiety^16,41^ and depression^16,41,42^ are strong prognostic factors for survival in lung cancer patients after treatment^39,44^. Posttraumatic growth factors also have good prognostic value^16^.

Although biomedical or clinical parameters are generally known as the first factors with prognostic value^45^^,^ HRQOL parameters have been regarded as additional values in predicting survival.^9,34,39^ However, even if we cannot change the clinical factors, HRQOL and lifestyle factors can be modified, therefore, we suggested HRQOL parameters as major effects to predict lung cancer survival. Better lung cancer prognostic indices based on both clinical and HRQOL factors need to be developed, and individual assessment algorithms for the prognosis of survivors are essential, guiding the clinical decision-making system to provide more information about their care based on MLTs. These HRQOL findings may indicate disease progression or recurrence that a physical examination by clinicians, a tumor marker evaluation, and imaging studies could not detect.^7^

In this study, MLTs were identified as having better predictive capabilities in clinical data sets than the traditional approach.^12^ Additionally, new ensemble learning-based prediction algorithms were more accurate than other MLTs. Although MLTs have been widely used to analyze gene expression data studies^46,47^ or medical image prediction analyses^48^, the studies that have explored MLTs in clinical settings are not sufficient, especially with respect to HRQOL. Our approach offers superior performance compared to previous machine learning approaches in predicting cancer survival. In addition, this approach could be used to better stratify lung cancer survivors in future clinical trials of cancer,improving the interpretation of study outcomes or helping identify critical areas could help in the selection of key endpoints for future clinical trials^49^.

Despite the superior performances of machine learning algorithms, it has been rather limited to use in routine clinical practices because such algorithms cannot be easily calculated with a traditional calculator. DT pruning and LR may produce predictive models with interpretable structures. RF and ensemble learning techniques, such as bagging or AdaBoost, are “black box” models^50–52^, where the function that links the response to the predictive variables is too opaque to use in daily clinical practice. One important advantage of the RF model is that the computational complexity inherent in support-vector machines (SVMs) can be reduced via quadratic optimization. Therefore, for convenience of use in clinical settings, developing a comprehensive digital-based self-management program by including a prediction model can provide more information and help survivors’ decisional support^53^.

However, our study has several limitations. First, there could be overfitting due to oversampling. It is obvious that the characteristics of the groups are clear, and it is not necessary to perform sampling. Since our data were imbalanced, which affects classification performance, it was necessary to balance the classes by sampling. When a classifier is correctly sampled, the classifier’s performance can be improved through oversampling. If the information in the existing data is lost or distorted during sampling, the learned algorithm does not properly reflect the characteristics of the original data. Therefore, data balancing using SMOTE-NC (non-continuous) to simulate the actual data, including noise that reflects the distribution of the existing data, should be employed in future studies. Second, MLTs that can be adapted to effectively handle survival data should be investigated^11^. The MLTs that we applied to this study cannot accurately predict the time of an event occurrence, and thus, we could not directly compare them with Cox-based prediction models. Previously, we performed the same process (using original imbalanced data) using Cox proportional hazard regression models, and the prediction model using HRQOL data in addition to clinical and sociodemographic variables was significantly better in terms of C-statistics (Supplementary Table 2). Therefore, including HRQOL with clinical variables together improved predictive performance in both traditional statistical analysis and machine learning techniques as well. Even though, because MLT didn’t consider the time of event, the cox-based model cannot be compared on the same lines. To handle survival problems with MLT, effective algorithms incorporate both statistical methods and MLT, such as survival trees^54^^,^ random survival forests^55^^,^ and bagging survival trees^28^. In addition, the participants were asked at different time intervals relative to the time of their diagnosis. Thus, at the fifth year from the date of survey completion after lung cancer surgery, we adjusted for this time difference by using a covariable that indicates a time since diagnosis. We suggest that an assessment of HRQOL data and lung cancer prediction models based on prognostic factors should be incorporated into routine clinical oncology practice, and further studies, such as randomized controlled trials, should be conducted. Although data from the study suggested that models adding HRQOL data were more accurate, this result should be validated in a wider population. Finally, due to a lack of other cohort sets including PROs among Korean lung cancer survivors, external validation was not conducted. Instead of an external validation set, the entire data set was randomly split to reduce the overfitting in the model then to produce a reliable estimate of the performance of the lung cancer survival model^6^. Future studies should validate the modeling process with other lung cancer cohort data including PROs.

The current study suggests that socio-clinical variables and HRQOL data can be applied to ensemble MLT algorithms (particularly the RF and AdaBoost algorithms) to predict disease-free lung cancer survival with better predictive performances than models using socioclinical variables only. Most importantly, including both HRQOL and lifestyle factors in a lung cancer survival prediction process with the RF model will provide patients with more accurate information and lower their decisional conflicts. Because cancer survivors need monitoring of multidimensional health-related problems^56^, the provision of appropriate information through a prediction model is important for better follow-up planning. The improved accuracy of MLT for lung cancer survival prediction can help clinicians and survivors make more clinically meaningful decisions about posttreatment care plans and their support in cancer care.

## Supplementary information

Supplementary information

## Data Availability

The data that support the findings of this study are available from the corresponding authors upon reasonable request.
