# Supplementary material for: The major effects of health-related quality of life on 5-year survival prediction among lung cancer survivors: applications of machine learning
Source: Sci Rep. 2020 Jul 1;10:10693. doi: 10.1038/s41598-020-67604-3 (PMC7329866; doi:10.1038/s41598-020-67604-3)
Supplement: Supplementary file 1 — Supplementary information [file 41598_2020_67604_MOESM1_ESM.docx]

**The major effects of health-related quality of life on 5-year survival prediction among lung cancer survivors: applications of machine learning**

Jin-ah Sim^1,2†^, Young Ae Kim^3†^, Ju Han Kim^4^, Jong Mog Lee^5^, Moon Soo Kim^5^, Young Mog Shim^6^, Jae Ill Zo^6,^ Young Ho Yun^1,4,7^*

^1^ Department of Biomedical Science, Seoul National University College of Medicine, Seoul, Korea

^2^ Department of Epidemiology and Cancer Control, St. Jude Children’s Research Hospital, TN, USA

^3^ National Cancer Control Institute, National Cancer Center, Goyang, Korea

^4^ Department of Biomedical Informatics, Seoul National University College of Medicine, Seoul, Korea

^5^Center for Lung Cancer, National Cancer Center, Goyang, Korea

^6^ Lung and Esophageal Cancer Center, Samsung Comprehensive Cancer Center, Samsung Medical Center, Seoul, Korea

^7^ Department of Family Medicine, Seoul National University College of Medicine, Seoul, Korea

* lawyun08@gmail.com

^†^ These authors equally contributed to this work as co-first authors.

Supplementary table 1. Final candidate variables from both literature review and statistical analyses

| Factors | Variables | Literature review evidence level | Significant variables form statistical analyses |
| --- | --- | --- | --- |
|  |  |  |  |
| Health Condition | Cancer stage | Strong | O |
|  | Local invasion of tumor | Strong | O |
|  | Regional Lymph node metastasis | Strong | O |
|  | Number of comorbidities | Strong | NS |
| Environmental Factors | Time since diagnosis | Weak | NS |
|  | Type of treatment | Strong | NS |
|  | Private insurance | Weak | NA |
|  | Low household income | Weak | O |
| Personal Factors | Age | Strong | O |
|  | Sex or Gender | Strong | O |
|  | Job status | Weak | Marginally significant |
|  | Education | Weak | NS |
|  | Marital status | Weak | NS |
| Body function and structures | BMI(kg/m2)  before operation | Strong | O |
|  | Anxiety | Strong | O |
|  | Depression | Strong | O |
|  | Physical functioning | Strong | O |
|  | Role functioning | Strong | O |
|  | Emotional functioning | Weak | NS |
|  | Cognitive functioning | Weak | NS |
|  | Social functioning | Weak | NS |
|  | General health QOL | Strong | NS |
|  | Fatigue | Strong | NS |
|  | Nausea and vomiting | Weak | NS |
|  | Pain | Weak | NS |
|  | Dyspnea | Strong | O |
|  | Appetite loss | Strong | O |
|  | Diarrhea | Weak | O |
|  | Constipation | Weak | NS |
|  | Lung cancer specific cough | Strong | O |
|  | Pain in chest | Strong | O |
|  | Peripheral neuropathy | Weak | NS |
|  | Post-traumatic growth | Weak | O |
|  | ECOG Performance | Strong | NA |
| Activities | Physical activity | Strong | O |
|  | smoking status | Weak | NS |
|  | Alcohol consumption | Weak | NS |
|  | Unhealthy dietary habits | Weak | NS |

**Abbreviation:** NS, Non-significant; NA, Not applicable; O, significant

Supplementary table 2. Validation of lung cancer survivors’ survival prediction models based on cox-proportional hazard regression models*

|  | |  |  | Increase in AUC (p) |
| --- | --- | --- | --- | --- |
|  | | Cox-1 Model | Cox-2 Model | Cox-2 Model  - Cox-1 Model |
| Development set | |  |  |  |
|  | C (95% CI) | 0.687  (0.649-0.725) | 0.797  (0.765-0.829) | 0.110 (<0.001) |
|  | Chi-square value  (p-value) | 11.883 (0.105) | 9.571  (0.297) |  |
| Validation set | |  |  |  |
|  | C (95% CI) | 0.699  (0.668-0.730) | 0.809  (0.783-0.835) | 0.1102 (<0.001) |
|  | Chi-square value  (p-value) | 2.793 (0.904) | 6.491 (0.592) |  |

* Stepwise-AIC best subsets approach in cox-regression were conducted.

Cox-1 Model includes sociodemographic and clinical variables: prediction score = 0.280 ⅹ (Age over 65 years (yes[1], no[0])) - 0.639 ⅹ (Female (yes[1], no[0])) + 0.306 ⅹ (Stage Ⅱ–Ⅲ (yes[1], no[0])) - 0.24 ⅹ (OP+RT Treatment (yes[1], no[0])) - 0.375 + 0.271ⅹ (Regional lymph node metastasis (yes[1], no[0]))

Cox 2 Model includes PRO variables and the variables from feature set 1**:** prediction score = -0.616 ⅹ (BMI (kg/m2) before operation ≥ 23 (yes[1], no[0])) + 0.788 ⅹ (Problematic role functioning (yes[1], no[0])) - 0.384ⅹ (Problematic dyspnea (yes[1], no[0])) + 0.802ⅹ (Personal strength < 15 (yes[1], no[0])) + 0.472 ⅹ (Appreciation of life < 18 (yes[1], no[0])) -0.746ⅹ (Female (yes[1], no[0])) + 0.275 ⅹ (Stage Ⅱ–Ⅲ (yes[1], no[0]))


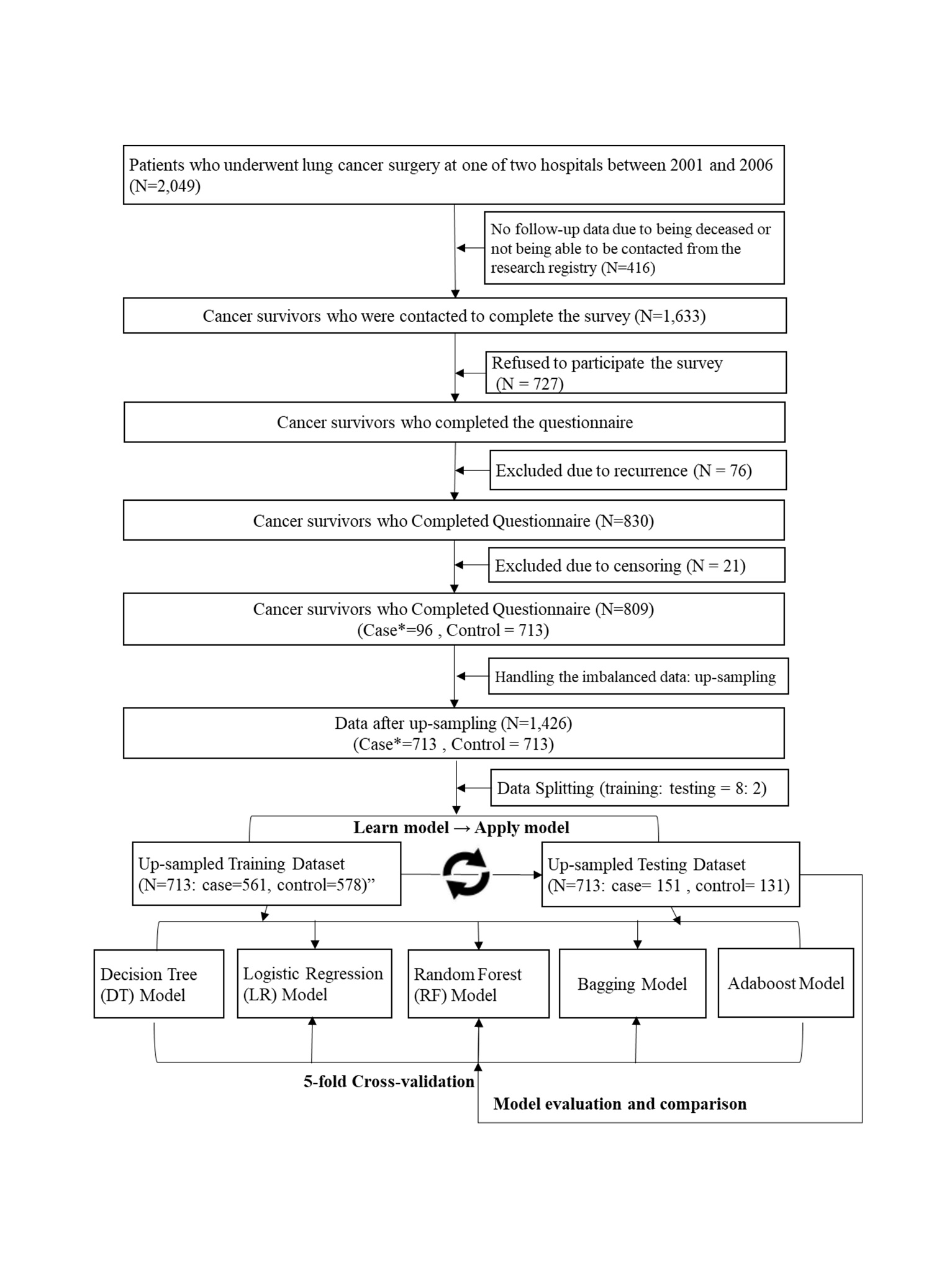


Supplementary Figure 1. Study flow chart

*Censored by lung cancer mortality within 5 years after completion of the primary lung cancer survey


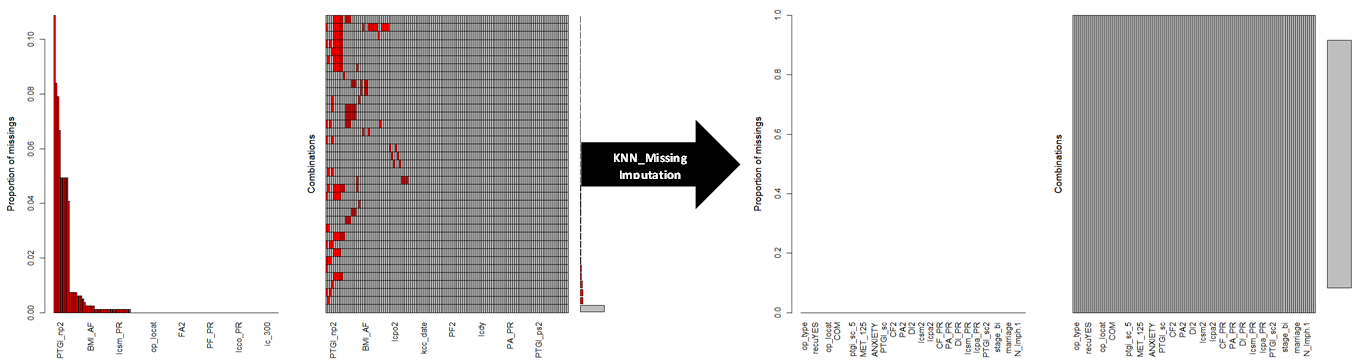


Supplementary Figure 2. Missing values change plots
